# Elucidating the Underlying Allelopathy Effects of *Euphorbia jolkinii* on *Arundinella hookeri* Using Metabolomics Profiling

**DOI:** 10.3390/plants14010123

**Published:** 2025-01-03

**Authors:** Xue Xiao, Zuyan Ma, Kai Zhou, Qiongmei Niu, Qin Luo, Xin Yang, Xiaohui Chu, Guilian Shan

**Affiliations:** Faculty of Animal Science and Technology, Yunnan Agricultural University, Kunming 650201, China; xiaoxue313507@163.com (X.X.); malbaixxi@163.com (Z.M.); zhoukai1988@126.com (K.Z.); 15887476442@163.com (Q.N.); luoqin268@gmail.com (Q.L.); 15288526540@139.com (X.Y.)

**Keywords:** *Euphorbia jolkinii*, allelopathy, photosynthesis, phytohormone, metabolomic, secondary metabolism, oxidative stress, plant stress responses

## Abstract

*Euphorbia jolkinii* dominates the subalpine meadows in Shangri-La (Southwest China) owing to its potent allelopathic effects. However, the effects underlying its allelopathy require further characterization at the physiological and molecular levels. In this study, the physiological, biochemical, and metabolic mechanisms underlying *E. jolkinii* allelopathy were investigated using *Arundinella hookeri* as a receptor plant. The treatment of *A. hookeri* seedlings with *E. jolkinii* aqueous extract (EJAE) disrupted their growth by inhibiting photosynthesis, disrupting oxidation systems, and increasing soluble sugar accumulation and chlorophyll synthesis. Collectively, this causes severe impairment accompanied by abnormal photosynthesis and reduced biomass accumulation. Moreover, EJAE treatment suppressed gibberellin, indoleacetic acid, zeatin, salicylic acid, and jasmonic acid levels while promoting abscisic acid accumulation. Further metabolomic analyses identified numerous differentially abundant metabolites primarily enriched in the α-linolenic, phenylpropanoid, and flavonoid biosynthesis pathways in EJAE-treated *A. hookeri* seedlings. This study demonstrated that *E. jolkinii* exhibits potent and comprehensive allelopathic effects on receptor plants, including a significant disruption of endogenous hormone synthesis, the inhibition of photosynthesis, an impairment of membrane and oxidation systems, and changes in crucial metabolic processes associated with α-linolenic, phenylpropanoid, and flavonoid biosynthesis. Thus, our study provides a solid theoretical foundation for understanding the regulatory mechanisms underlying *E. jolkinii* allelopathy.

## 1. Introduction

*Euphorbia jolkini* Boiss. is a perennial toxic plant widely distributed in the subalpine meadows of Shangri-La, in northwestern Yunnan [1]. It can expand beyond where it typically thrives due to its well-adapted, resistant, and advanced root system [1]. Due to this feature, the species has emerged as the dominant indigenous invasive species [2]. *E. jolkinii* forms a homogeneous community at the expense of various common species, such as *Arundinella hookeri* Munro ex Keng and *Carex tristachya* Thunb. [3]. This phenomenon has been attributed to its strong competitiveness and allelopathic effects on native species [4]. *A. hookeri* is a perennial herb of the Gramineae family that serves as an important natural forage within northwestern Yunnan. Hence, the loss of *A. hookeri* and the expansion of *E. jolkinii* has severely impacted the sustainable development of local animal husbandry.

Allelopathy is the phenomenon by which plants release secondary metabolites, known as allelochemicals, into the environment to influence the growth of neighboring plants directly or indirectly [5,6]. Allelochemicals can directly interfere with the photosynthesis, respiration, enzyme synthesis, and metabolism of target plants [7] or indirectly through soil fertility and beneficial soil microorganisms that affect plant communities [8]. *E. jolkinii* releases various chemical compounds into the soil primarily through root exudation and the decomposition of plant residues [9]. These compounds, including phenolic acids, amino acids, lipids, flavonoids, alkaloids, and terpenoids, are spread through the soil by rain leaching [10]. Among these compounds, 4-nitrophenol, quinic acid, and 2-phenylethylamine have significant inhibitory effects on seed germination and the fresh weights, plant heights, and root lengths of *Lolium perenne* L. and *Trifolium pratense* L. [11]. Additionally, meta-tyrosine from *E. jolkinii* exhibits significant concentration-dependent inhibitory effects on root and shoot growth in *Zea mays* L., *Medicago sativa* L., and *Brassica campestris* L. [12]. This inhibition is primarily achieved by reducing the root activity and increasing the malondialdehyde content, blocking gibberellin (GA), zeatin (ZT), and jasmonic acid (JA) synthesis, consequently, inhibiting seedling growth [3,4]. However, studies on the allelopathic effects of *E. jolkinii* have focused primarily on seed germination, seedling growth, and physiological and biochemical parameters of the receptor plants. Meanwhile, the molecular effects underlying allelopathy remains unknown.

In this study, we aimed to explore the allelopathic effects of the aqueous extracts of *E. jolkinii* on *A. hookeri* using physiological and metabolomic analysis. Specifically, we evaluated seedling growth, photosynthetic physiology, alterations in endogenous hormone levels, and differential accumulated metabolites (DAMs) in receptor plant seedlings. The results of this study provide valuable insights into the regulatory effects underlying allelopathy that can inform the development of conservation and biocontrol measures to prevent *E. jolkinii* invasion.

## 2. Results

### 2.1. Allelopathic Effects of EJAE on A. hookeri Seedling Growth

**Figure 1:**
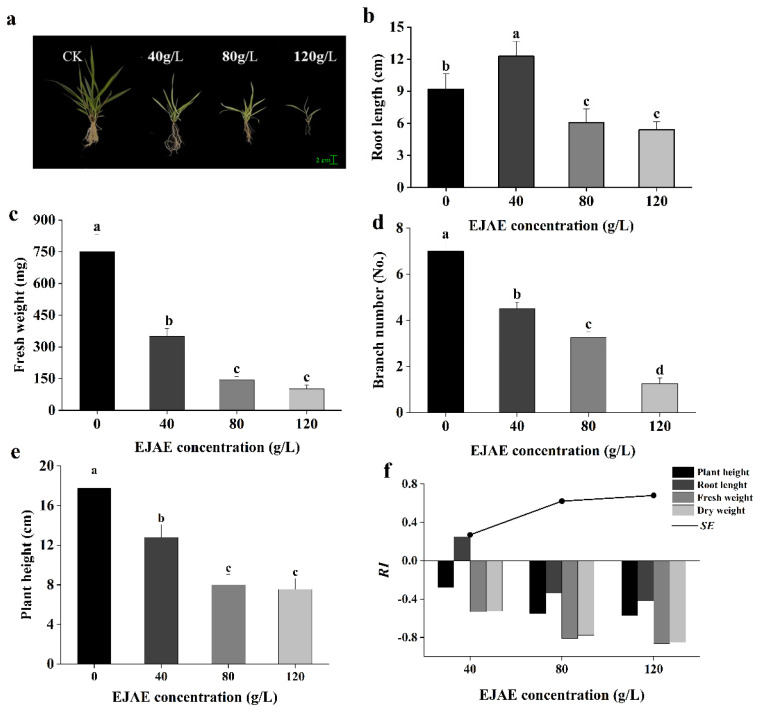
Effects of EJAE treatment on *A. hookeri* (*Arundinella hookeri* Munro ex Keng) seedlings. (**a**) *A. hookeri* seedlings treated with different concentrations of EJAE, (**b**) root length, (**c**) fresh weight, (**d**) branch number, (**e**) plant height, (**f**) *RI*, and *SE*, respectively. Data are presented as the means ± standard deviations (n ≥ 3 biological replicates). Lowercase letters: using the one-way ANOVA (Duncan), indicating significant differences (*p* < 0.05) between 0 (control), 40 (treatment with 40 g/L EJAE), 80 (treatment with 80 g/L EJAE), and 120 (treatment with 120 g/L EJAE) groups.

Overall, *A. hookeri* seedling growth was significantly inhibited by EJAE in a concentration-dependent manner (Figure 1a). The root length showed a phenomenon of stimulation at low concentrations and inhibition at high concentrations of EJAE. Although a significant increase in the root length was observed at the 40 g/L EJAE concentration compared with the control treatment, when the concentration was >40 g/L, a significant decrease was observed in the root length (*p* < 0.001; Figure 1b). Compared with the control treatment, the fresh weight, branch number, and plant height of *A. hookeri* seedlings significantly decreased under EJAE treatment (*p* < 0.001; Figure 1c–e). Additionally, *A. hookeri* leaves turned yellow under 80 and 120 g/L EJAE treatment conditions. The response index (*RI*) and synthetic allelopathic effect index (*SE*) results (Figure 1f) illustrated that EJAE had an inhibitory effect on seedling growth. These findings suggest that EJAE suppresses the growth of *A. hookeri* seedlings.

### 2.2. Allelopathic Effects of EJAE on Photosynthesis in A. hookeri Seedlings

**Figure 2:**
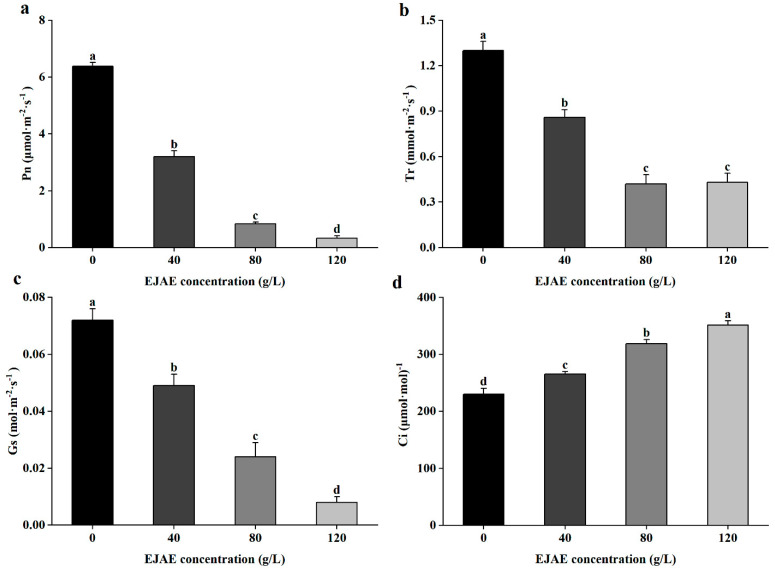
Effects of EJAE treatment on photosynthesis in *A. hookeri* seedlings. (**a**) Pn, (**b**) Tr, (**c**) Gs, and (**d**) Ci. Data are presented as the means ± standard deviations (n ≥ 3 biological replicates). Lowercase letters: using the one-way ANOVA (Duncan), indicating significant differences (*p* < 0.05) between 0 (control), 40 (treatment with 40 g/L EJAE), 80 (treatment with 80 g/L EJAE), and 120 (treatment with 120 g/L EJAE) groups.

The photosynthetic rate (P_n_), transpiration rate (T_r_), and stomatal conductance (G_s_) in the treatment groups concomitantly decreased, which caused the biomass of the *A. hookeri* seedlings to significantly decrease in response to EJAE treatment. In contrast, the intercellular carbon dioxide concentration (C_i_) significantly increased compared with the control group (Figure 2a–d). For example, under EJAE-120, P_n_, T_r_, and G_s_ decreased by 94.8%, 66.9%, and 88.9%, respectively, compared with the corresponding levels in the control group (Figure 2a–c). Conversely, C_i_ increased by 52.5% (Figure 2d). Changes in the P_n_, T_r_, and G_s_ showed effects on *A. hookeri* seedling photosynthesis.

### 2.3. Allelopathic Effects of EJAE on the Chlorophyll Content and Antioxidative Systems in A. hookeri Seedlings

**Figure 3:**
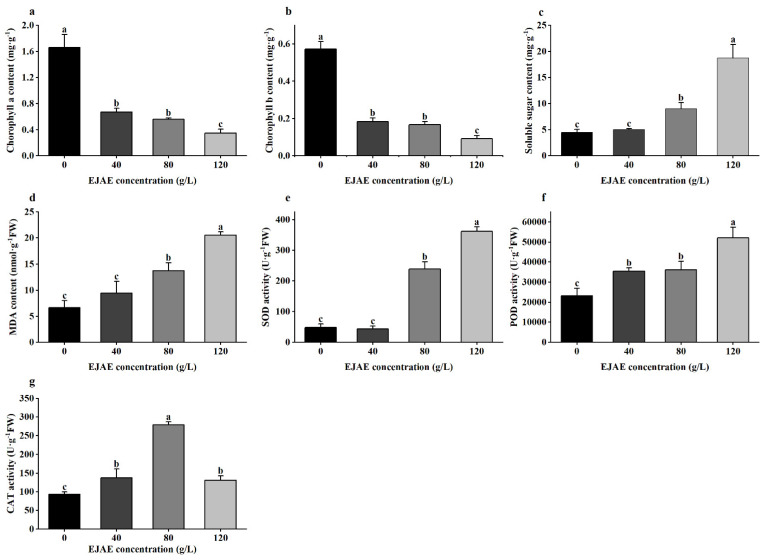
EJAE treatment disrupts *A. hookeri* seedling growth and causes oxidative damage. (**a**) Chlorophyll a and (**b**) chlorophyll b contents. (**c**) Soluble sugar content. (**d**) MDA content. (**e**) SOD, (**f**) POD, and (**g**) CAT activities. FW in (**d**–**g**) means fresh weight. Data are presented as the means ± standard deviations (n ≥ 3 biological replicates). Lowercase letters: using the one-way ANOVA (Duncan), indicating significant differences (*p* < 0.05) between 0 (control), 40 (treatment with 40 g/L EJAE), 80 (treatment with 80 g/L EJAE), and 120 (the treatment with 120 g/L EJAE) groups.

Compared with the control group, a significant concentration-dependent decrease in the chlorophyll content in *A. hookeri* leaves treated with EJAE-40, EJAE-80, and EJAE-120 was observed (Figure 3a,b). Leaf Chl-a and Chl-b contents under the 120 g/L EJAE treatment accounted for only 24.1% and 16.1% of those of the control group, respectively. In turn, the soluble sugar content significantly increased (Figure 3c) in response to *E. jolkinii* in all three treatment groups, with EJAE-120 showing a 4.1-fold increase compared with the control group.

The levels of MDA increased as the concentration of EJAE increased (Figure 3d). Following EJAE-120, the MDA content was 210% higher than that of the control group. Similarly, the activities of superoxide dismutase (SOD), peroxidase (POD), and catalase (CAT) were significantly affected by *E. jolkinii* allelopathy (Figure 3e–g). Particularly, the SOD and POD activities in the treatments were significantly (*p* < 0.001) higher (approximately 655.70% and 124.10%, respectively) than in the control (Figure 3e–g). Moreover, although the CAT activity tended to increase in the EJAE-40 and EJAE-80 treatments, it decreased under EJAE-120 (Figure 3g).

### 2.4. Allelopathic Effects of EJAE on Phytohormone Homeostasis in A. hookeri Seedlings

**Figure 4:**
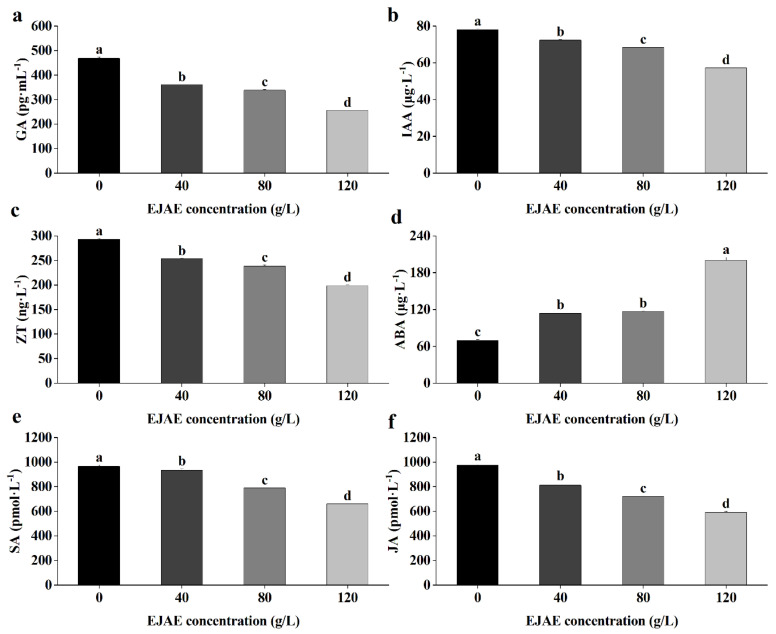
EJAE treatment alters phytohormone homeostasis in *A. hookeri* seedlings. (**a**) GA, (**b**) IAA, and (**c**) ZT contents, respectively. Stress-responsive phytohormone contents: (**d**) ABA, (**e**) SA, and (**f**) JA. Data are presented as the means ± standard deviations (n ≥ 3 biological replicates). Lowercase letters: using the one-way ANOVA (Duncan), indicating significant differences (*p* < 0.05) between 0 (control), 40 (treatment with 40 g/L EJAE), 80 (treatment with 80 g/L EJAE), and 120 (treatment with 120 g/L EJAE) groups.

The effect of EJAE on endogenous phytohormone production in *A. hookeri* seedlings was evaluated. The results revealed that EJAE-40 effectively suppressed GA and indole acetic acid (IAA) production. Meanwhile, compared with that of the control group, the GA content decreased by 45.30%, whereas the IAA content decreased by 26.71% under EJAE-120. (Figure 4a,b). A significant decrease was also observed in the ZT content with an increasing EJAE concentration. Particularly, when the extract concentration reached 120 g/L, the ZT content was only approximately 67.60% of that of the control group (Figure 4c).

Regarding stress-responsive phytohormones, including abscisic acid (ABA), salicylic acid (SA), and JA, EJAE treatment significantly increased the ABA levels compared with the control group. Particularly, the ABA content was 189.1% higher under EJAE-120 than in the control group (Figure 4d). Conversely, a decreasing trend was observed for the SA and JA contents with increasing EJAE concentrations (Figure 4e,f).

### 2.5. Allelopathic Effects of EJAE on the Metabolite Composition of A. hookeri Seedlings

**Figure 5:**
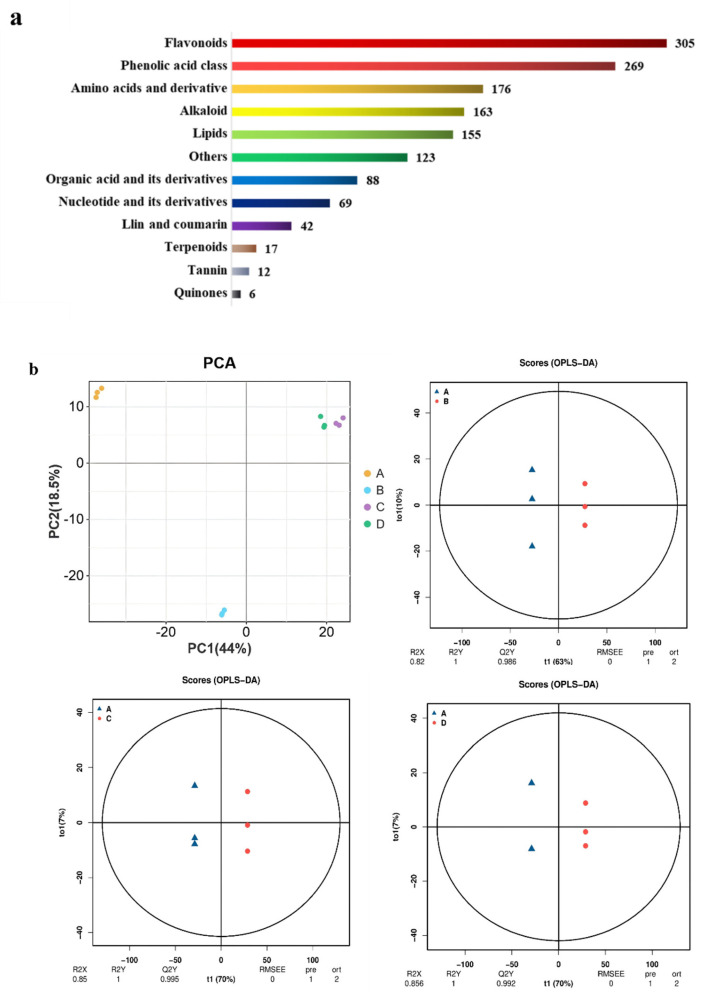
Metabolome of the allelopathic inhibitory effect of EJAE on *A. hookeri* seedlings. (**a**) Classification of the 1425 identified metabolites. (**b**) PCA of the identified metabolites and OPLS-DA scores (A, the control group; B, EJAE-40; C, EJAE-80; D, EJAE-120).

Through metabolomic analysis, 1425 metabolites (Supplementary Table S1) belonging to 12 distinct chemical categories were identified in the *A. hookeri* seedlings (Figure 5a). The predominant categories included flavonoids, phenolic acids, amino acids, and alkaloids, which collectively accounted for 64% of all detected metabolites. Principal component analysis (PCA) and a heat map depicting the metabolomic profiles revealed clear variations among treatments, with high reproducibility across replicates (Figure 5b).

### 2.6. DAMs and Pathways Among Different EJAE Treatments

**Figure 6:**
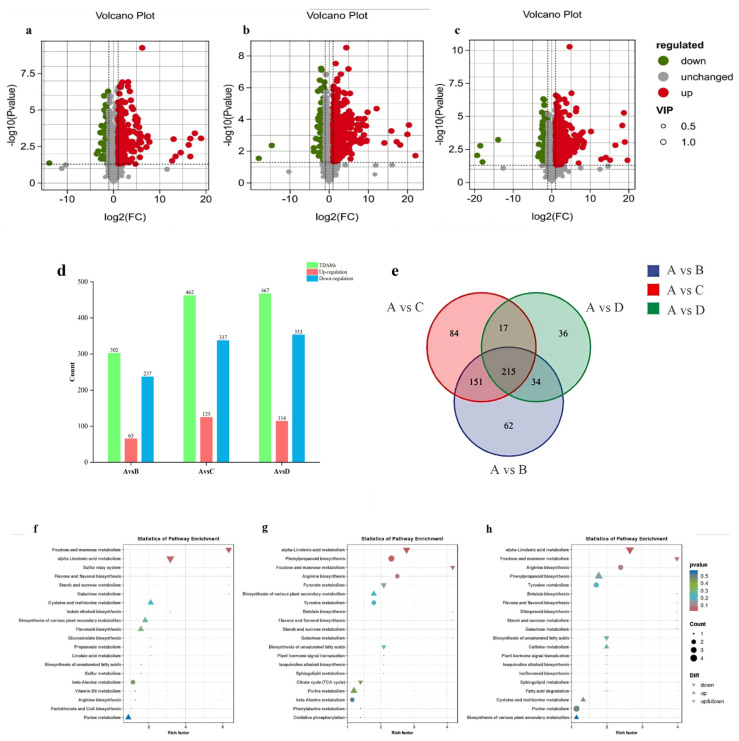
Changes in metabolites resulting from EJAE treatment occur in various pathways. (**a**–**c**) Volcano plots of DAMs in different treatment groups versus the control group (A represents the control group vs. EJAE-40, B represents the control group vs. EJAE-80, and C represents the control group vs. EJAE-120). (**d**) Upregulated and downregulated DAMs in different comparison groups (TDAMs: total differential accumulated metabolites). (**e**) Venn diagrams: each circle represents a comparison group, with overlapping regions representing the common metabolites and nonoverlapping regions representing the unique metabolites within groups. Top 20 enriched metabolic pathways associated with the DAMs in (**f**) EJAE-40, (**g**) EJAE-80, and (**h**) EJAE-120 groups versus the control group. Capital letters A, B, C, and D in Figure (**e**) represent the control group and EJAE-40, EJAE-80, and EJAE-120 treatments, respectively.

To identify DAMs between the different EJAE and control groups, three thresholds were applied: importance in project (VIP) > 1, fold change (FC) > 2 or <0.5, and *p* < 0.05. Volcano plots, histograms, and Venn diagrams were generated to visualize all DAMs under EJAE-40 compared with the control (A represents the control group versus (vs.) EJAE-40; 237 upregulated and 65 downregulated), in the EJAE-80 compared with the control group (B represents the control group vs. EJAE-80; 337 upregulated and 125 downregulated), and in the EJAE-120 compared with the control group (C represents the control group vs. EJAE-120; 353 upregulated and 114 downregulated) (Figure 6a–e, Supplementary Tables S2–S4).

The Kyoto Encyclopedia of Genes and Genomes (KEGG) analysis revealed that the allelopathic interaction influenced multiple biological pathways (Figure 6f–h). Particularly, α-linolenic acid metabolism was downregulated in response to EJAE treatment. Meanwhile, phenylpropanoid and flavonoid biosynthesis was upregulated with an increasing EJAE concentration. Metabolites released in response to allelopathy under EJAE-80 and EJAE-120 treatments showed a higher level of specific enrichment in the phenylpropanoid biosynthesis category than in the control or EJAE-40 treatment groups. These findings demonstrate significant EJAE concentration-dependent changes in metabolite composition and metabolic pathway enrichment.

**Figure 7:**
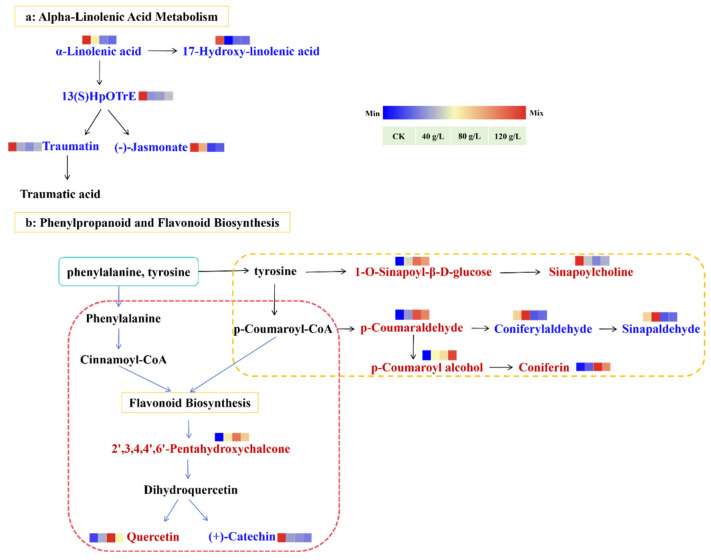
Analysis of critical metabolic pathways associated with allelopathic inhibition in *A. hookeri*. (**a**) α-Linolenic acid metabolism; (**b**) phenylpropanoid and flavonoid biosynthesis. Red, blue, and black colors indicate that the metabolite content was significantly upregulated, downregulated, or did not significantly differ, respectively.

A diagram was constructed to better understand the distribution of metabolites within the biosynthetic pathways of α-linolenic acid, phenylpropanoids, and flavonoids (Figure 7). With respect to α-linolenic acid metabolism, 17-hydroxy-linolenic acid, 13(S)-HpOTrE, traumatin, and (-)-jasmonate were downregulated. This downregulation of (-)-jasmonate was consistent with the hormone levels recorded in this study.

Regarding the phenylpropanoid and flavonoid biosynthesis pathways, a close association was observed between the phenylpropanoid and flavonoid biosynthesis pathways and their metabolites. The flavonoid biosynthesis pathway was activated and upregulated under EJAE-40. However, as its concentration increased, the enrichment of the flavonoid biosynthesis pathway diminished, whereas the phenylpropanoid biosynthesis pathway was upregulated.

## 3. Discussion

### 3.1. EJAE Causes Oxidative Damage in A. hookeri

Reactive oxygen species are inevitable byproducts of bioenergetic processes that occur via electron transport [13,14]. Their excessive accumulation can disrupt cellular homeostasis and compromise organelle integrity under environmental stress conditions [15,16]. Therefore, plants have developed endogenous antioxidant enzyme defense systems to counteract oxidative stress induced by various factors, including allelopathic interactions [17,18]. These defense systems comprise antioxidant enzymes, such as SOD, POD, and CAT, whose ROS-scavenging activity safeguards cellular membrane phospholipids against peroxidation [19]. As the final product of lipid peroxidation associated with oxidative stress, MDA serves as an indicator of cellular injury, particularly membrane damage [20,21]. The allelopathic effects of EJAE resulted in a burst of ROS and a breakdown of the cellular membrane system. At low EJAE concentrations, the SOD, POD, and CAT activities were enhanced to maintain relatively stable ROS levels. However, as the concentration increased, the MDA content increased, which may result in cell damage as suggested by Nicolas et al. [22]. Consequently, oxidative damage is an important factor by which the allelopathy inhibits the growth of receptor plants. This is consistent with the allelopathic effects of *Callistemon viminalis* on wheat and chickpea plants [23].

### 3.2. EJAE Caused Photosynthetic and Metabolic Disorders Resulting in “Starvation” of A. hookeri Seedlings

In general, seedling growth requires photosynthesis for the production of organic matter to fuel plant growth and development by absorbing light energy, carbon dioxide, and water [24]. Our results indicate that one of the critical effects underlying allelopathy seemingly involves the induction of “starvation” in *A. hookeri* seedlings by disrupting photosynthesis and primary metabolism. This aligns with the results of Li et al. [25] on the allelopathic effects of *Artemisia argyi* H. Lév. & Vaniot. Significantly reduced P_n_, T_r_, and G_s_ rates and chlorophyll contents were observed under EJAE treatment, whereas the C_i_ content increased. These changes suggest severe damage to the chloroplasts and cell membranes, resulting in a cascade of effects that further decreased the biomass of *A. hookeri* seedlings and severely inhibited their growth [25].

Additionally, metabolomic analysis revealed a significant downregulation of metabolites and metabolic pathways associated with carbohydrate metabolism, including fructose and mannose metabolism, pyruvate metabolism, and the citrate cycle. These results provide further evidence that allelopathy by *E. jolkinii* significantly impedes biomass accumulation, growth, and the development of receptor plants.

Endogenous hormone regulation is crucial for plant growth, development, metabolism, and environmental responses [26]. In *A. hookeri* seedlings, endogenous hormone regulation was significantly disrupted following EJAE treatment. Our results showed that increasing concentrations of EJAE increased the ABA levels and reduced the GA, IAA, ZT, SA, and JA levels. Together with metabolomic results, the evidence suggests that the plant hormone signal transduction pathway was significantly downregulated. The pyruvate pathway, which serves as a fundamental and primary route for auxin synthesis in plants [27], was downregulated, indicating that allelopathy induced by *E. jolkinii* modified the hormone pathway in *A. hookeri*, resulting in a substantial reduction in auxin levels. Allelopathy can disrupt hormone balance and affect normal cell division, elongation, and differentiation processes, and these abnormal changes hinder the efficiency of root physiological functions, such as absorbing and transferring nutrients [28,29]. Therefore, “starvation” of the receptor plants may be another key effect of allelopathy. Taken together with the results of previous research, we found that inducing hormone disorders resulted in metabolic imbalances and hindered the uptake of essential nutrients, thereby accelerating receptor plant death [25].

Metabolomic analysis results revealed a significant enrichment of secondary metabolic pathways in *A. hookeri*, indicating its ability to resist allelopathic stress caused by EJAE. This is consistent with previous research demonstrating that plants undergo a shift from primary to secondary metabolism under abiotic and biotic stress conditions, limiting biomass accumulation and impeding growth [30]. The α-linolenic acid, phenylpropanoid, and flavonoid pathways play vital roles in allelopathic responses during seedling growth. Whittaker et al. [31] indicated that the phenylpropanoid and flavonoid pathways are intricately linked to allelopathy. Phenylpropanoid metabolism significantly contributes to plant development and plant–environment interactions [32]. For example, phenylpropanes, such as lignin, flavonoids, phenylpropanoid esters, hydroxycinnamic acid amides, and sporopollenin, are well-known secondary metabolites that inhibit the growth and development of neighboring plants [33,34]. Similarly, flavonoids, including flavones, flavanols, and flavanones, show diverse allelopathic activities and contribute to the regulation of plant–plant interactions [35,36,37]. These findings correspond with the allelopathic interactions between rice and the invasive weed *Leptochloa chinensis* (L.) Nees [38]. In this study, the observed enrichment suggests that *A. hookeri* responded to allelopathy by regulating phenylpropanoid and flavonoid synthesis. Furthermore, the α-linolenic acid metabolic pathway serves as a crucial signaling pathway in plant-induced defense responses and is closely associated with JA synthesis [39]. The enzymes responsible for initiating JA synthesis from linolenic acid not only play an essential role in JA production but also serve as important signaling molecules in plant-induced defense responses [40,41].

**Figure 8:**
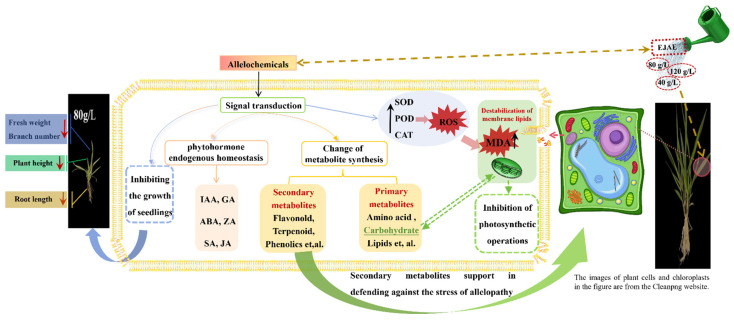
Effect of EJAE allelopathy on various physiological and metabolic functions in *A. hookeri* seedlings.

The results of this study clearly showed that allelopathy by EJAE had comprehensive effects on the receptor plants, including a large-scale endogenous hormone synthesis imbalance, photosynthesis inhibition, damage to the membrane and oxidation systems, and other pivotal metabolic processes in *A. hookeri* (Figure 8). Furthermore, the allelopathic effects of EJAE on receptor plants are likely caused by the allelochemicals, such as, 4-nitrophenol, quinic acid, and 2-phenylethylamine, which exert significant inhibitory effects on *L. perenne* and *T. pratense* [13]. Meta-tyrosine shows significant concentration-dependent inhibitory effects on root and shoot growth in *Z. mays*, *M. sativa*, and *B. campestris* [12]. These findings provide novel insights into allelopathic effects in plant stress responses, providing new perspectives for understanding allelopathy and allelochemicals.

## 4. Materials and Methods

### 4.1. Plant Material

After cultivation for over three years, a mature *Euphorbia jolkini* Boiss. plant was collected in October 2021 in Shangri-La, Yunnan Province, China (27°29′0″ N, 99°52′16″ E) and taxonomically identified by Professor Guilian Shan from Yunnan Agricultural University. Voucher specimens (20211001 and 20211002) were deposited in the herbarium of Yunnan Agricultural University.

Following collection, the vegetative parts of *E. jolkinii* were meticulously separated, thoroughly washed, air-dried, and used to prepare the extracts. *Arundinella hookeri* Munro ex Keng seeds were collected from Shangri-La in October 2021. After collection, the seeds were air-dried before use in a pot experiment.

### 4.2. Preparation of the E. jolkinii Aqueous Extract (EJAE)

The vegetative parts of *E. jolkinii* show a higher abundance of secondary metabolites than the roots and exert a stronger allelopathic inhibitory effect [10,11]. Therefore, in this study, we used the vegetative parts of *E. jolkinii* to prepare the EJAE, according to the procedure described by Yu et al. [42] after some modifications. The air-dried vegetative parts were pulverized and sieved. A total of 120 g of the powdered material was weighed, added to 1000 mL of ultrapure water, and subjected to separation in a thermostatic oscillator (Shanghai Jinghong Experimental Equipment Co., Ltd., Shanghai, China) at 25 °C for 12 h. The mixture was extracted for another 12 h and filtered using gauze and a notch filter (Tianjin Jinteng Experimental Equipment Co., Ltd., Tianjin, China) (0.45 μm) to obtain an EJAE stock solution (120 g/L), which was stored at 4 °C for future use. Three concentrations of EJAE (120, 80, and 40 g/L) were used: 120 g/L was the stock solution, whereas 80 and 40 g/L were prepared by diluting the stock solution. The selection of these three concentrations was based on our previous research [4].

### 4.3. Allelopathic Effect of EJAE on A. hookeri

#### 4.3.1. Cultivation of *A. hookeri* Seedlings

*A. hookeri* seedlings were cultivated using the seedling tray method [43]. According to our preliminary experiments [4], six-hole trays with 150 mL holes were prepared with a well-balanced 1:1 mixture of perlite and vermiculite. After disinfection, 20 seeds were sown into a hole. The trays were then placed in a climate cabinet (Shanghai YIHENG Scientific Instrument Co., Ltd., Shanghai, China) at 25 ± 1 °C under photoperiod and light intensity regimes of 14 h light/10 h dark and 6000xl, respectively [44]. A Hoagland nutrient solution (Shanghai yuanye Bio-Technology Co., Ltd., Shanghai, China) was applied to irrigation every other day at 30 mL per hole during germination. One tray constituted one repetition, and three repetitions were set for each treatment.

After germination, seedlings were planted uniformly in trays, with five plants per hole; each treatment was applied to three six-hole trays [45]. After 4 weeks of growth, the seedlings were watered every 3 days with 20 mL of 40, 80, or 120 g/L EJAE, respectively, and water with Hoagland nutrient solution (Shanghai yuanye Bio-Technology Co., Ltd., Shanghai, China) one day after applying the EJAE. The three treatment groups were labeled EJAE-40, EJAE-80, and EJAE-120. Distilled water was used as the control treatment. The growth conditions for the seedlings in the climate cabinet (Shanghai YIHENG Scientific Instrument Co., Ltd., Shanghai, China) were consistent with those during the seed germination phase. After 21 days of irrigation, morphological, photosynthetic, physiological, and endogenous hormone indicators were measured.

#### 4.3.2. Analysis of *A. hookeri* Seedling Growth

The plant height, root length, fresh weight, dry weight, and branch number were measured according to the methods of Fan et al. [46], with slight modifications. For each concentration treatment, 10 seedlings with robust growth were selected for individual measurements. The plant height was measured as the distance from the junction of the roots and stems to the tip of the plant. The root length was measured as the distance from the junction of roots and stems to the basal portion of the primary root. Water on the leaves was blot-dried using absorbent paper, and the fresh weight was measured with a 1/10,000 electronic balance (Cubis II, Sartorius, Göttingen, Germany). After measuring the fresh weight, the plants were dried to a constant weight at 65 °C (dry weight).

#### 4.3.3. Analysis of *A. hookeri* Seedling Photosynthetic Performance

Leaf T_r_, P_n_, C_i_, and G_s_ were measured from 8:30 to 11:30 on sunny mornings using a Li-6400 Portable Photosynthesis System (Lincoln, NE, USA), as described by Wang et al. [47]. The light intensity, temperature, relative humidity, and carbon dioxide concentration were set to 400 mol/(m^2^·s), 25 °C, 65%, and 300 μmol/mol, respectively. Three seedlings were randomly selected from each treatment group, and three different leaves from each seedling were selected for measurement. Data are presented as the means of measurements for the upper, middle, and lower portions of three leaves in each replicate according to the method described by Chen et al. [48].

#### 4.3.4. Analysis of *A. hookeri* Leaf Chlorophyll Content

The spectrophotometric approach was used to determine the amount of chlorophyll a (Chl-a) and b (Chl-b) [49]. Undamaged and different parts of the leaves from each treatment for measurement were randomly selected. Three repeats were set for each treatment. Leaf tissue samples (1.0 g) were finely ground into a powder under liquid nitrogen using a mortar and pestle (Shanghai Precision Instrument Co., Ltd., Shanghai, China). Subsequently, 5 mL of 80% cold acetone (Lihua Yiwei Yuan Chemical Co., Ltd., Dongying, China) was added to the tissue powder and incubated at 4 °C for 3 h. After incubation, the mixture was centrifuged at 6000× *g* for 15 min. Following centrifugation, 1 mL of the supernatant was transferred to a quartz cuvette, and the absorbances at 663 nm and 645 nm were measured using a spectrophotometer (A560, AOE Instruments, Co., Ltd., Shanghai, China). The concentrations of chlorophyll a and chlorophyll b were determined using the equations provided by Hiscox et al. [50].

#### 4.3.5. Analysis of *A. hookeri* Seedling Antioxidative Systems

The antioxidative responses of seedlings were analyzed using the corresponding commercial enzyme kits, following the protocols recommended by the manufacturers. Briefly, the vegetative parts of three seedlings from the different treatment groups were randomly selected, finely chopped, and mixed uniformly. Three repeats were set for each treatment. Then, less than 0.1 g of leaf tissue was homogenized with 1 mL of extraction solution in an ice bath [25,51]. The mixture was centrifuged at 12,000× *g* for 10 min at 4 °C, and the MDA content, SOD, CAT, and POD activities were assessed in the supernatants.

In turn, 0.1 g of leaf tissue was homogenized with 1.5 mL of 80% ethyl alcohol (Lihua Yiwei Yuan Chemical Co., Ltd., Dongying, China) in an ice bath [51]. The mixture was heated for 20 min in a 50 °C water bath and centrifuged for 10 min at 12,000× *g*. The supernatant was used to determine soluble sugar content.

Soluble sugar and MDA contents were determined using commercial kits following the instructions of the manufacturer (Suzhou Grith Biotechnology Co., Ltd., Suzhou, China). Similarly, the SOD, POD, and CAT activities were assayed using commercial kits as recommended by the manufacturer (Nanjing Jiancheng Bioengineering Institute, Nanjing, China).

#### 4.3.6. Analysis of *A. hookeri* Seedling Phytohormone Contents

The GA, ABA, IAA, ZT, SA, and JA levels were measured using enzyme-linked immunosorbent assays [52] with commercial assay kits as per the instructions provided by the manufacturer (Nanjing Jiancheng Bioengineering Institute). The vegetative parts of three seedlings from different treatment groups were randomly selected, finely chopped, and mixed uniformly for measurement. Three repeats were set for each treatment. Absorbance was determined using a visible light spectrophotometer (A560, AOE Instruments (Shanghai) Co., Ltd., Shanghai, China).

#### 4.3.7. Analysis of Secondary Metabolites from *A. hookeri* Seedlings

The vegetative parts of three seedlings from different treatments were randomly selected and used to determine secondary metabolites using Ultra-Performance Liquid Chromatography–Tandem Mass Spectrometry (UPLC-MS) technology. UPLC and qualitative analyses of the secondary metabolites detected were performed according to the protocol described by Liu et al. [45].

Metabolite extraction and profiling were performed as previously described [45]. Samples were freeze-dried using a lyophilizer (SCIENTZ-10N/A, Ningbo Xinzhi Biotechnology Co., Ltd., Ningbo, China), and ground to a powder. Next, 100 mg of powder was dissolved in 1.2 mL of 70% methanol (Lihua Yiwei Yuan Chemical Co., LTD, Dongying, China) and vortexed for 30 s once every 30 min six times. The samples were refrigerated at 4 °C overnight, and then the filtered samples were analyzed using an ultra-high-performance liquid chromatograph (UHPLC) coupled to a quadrupole time-of-flight mass spectrometer (qToF MS, Bruker Compact, Karlsruhe, Germany) with electrospray ionization. A broad-targeted analysis of metabolites was performed.

An Agilent SB-C18 (1.8 μm, 2.1 mm × 100 mm) chromatographic column was used. UPLC conditions were as follows: mobile phase A comprised 0.1% formic acid in water, while mobile phase B was acetonitrile. The gradient elution was as follows: 0 min, 5% B; 9 min, 95% B; 10–11 min, 5% B; and equilibrates at 5% to 14 min. The column temperature was maintained at 40 °C.

Linear ion trap (LIT) and triple quadrupole (QQQ) scans were acquired on a triple quadrupole–linear ion trap mass spectrometer (QTRAP), QTRAP LC-MS/MS System, equipped with an electrospray ionization (ESI) Turbo Ion-Spray interface, operating in positive and negative ion modes and controlled using Analyst 1.6.3 software (Sciex). ESI source conditions were set as follows: ion spray voltage, +5500/−4500 V; gas curtain, 35 psi; temperature, 600 °C; gases 1 and 2 at 60 psi for the ion source; and DP, ±100 V.

Metabolite abundances were quantified using the peak areas. Data obtained from metabolite profiling were analyzed using MetaboAnalystR. The identification of metabolites was based on their precise mass, MS2 fragment patterns, isotopic distributions in MS2 fragments, and retention times (RTs). Using a secondary spectrum-matching method, the secondary spectra and RTs of metabolites in the sample were intelligently matched against those in the company’s (Biomarker Biotechnology Co., Ltd., Beijing, China) database, GB-PLANT, constructed using standard samples, public databases, and references, along with manual identification by experts. PCA was performed, and a partial least-squares discriminant analysis (PLS-DA) model was built to obtain VIP values. Metabolites were defined as significant DAMs when the VIP > 1, FC > 2 (upregulated) or < 0.5 (downregulated), and *p*-value < 0.05 [53]. The identified metabolites were annotated using the KEGG Compound database (http://www.kegg.jp/kegg/compound/, accessed on 10 October 2022), and the annotated metabolites were mapped to the KEGG Pathway database (http://www.kegg.jp/kegg/pathway.html, accessed on 10 October 2022). Subsequently, pathways exhibiting significantly regulated metabolites were subjected to metabolite set enrichment analysis (MSEA). The significance of these pathways was assessed using hypergeometric tests [54].

### 4.4. Statistical Analysis

All the data were analyzed using SPSS software (SPSS 23.0, IBM, Armonk, New York, NY, USA) and are expressed as means ± standard deviations. The homogeneity of variance and normality were respectively tested before the analysis of variance (ANOVA) was conducted. The normality of data was checked using the Shapiro–Wilk test and Q–Q plots test. Subsequently, ANOVA and Duncan’s multiple comparisons were performed for each group. Statistical significance was set at *p* < 0.05. Graphs were drawn using Excel (Microsoft Excel 2021, Microsoft, Redmond, Washington, DC, USA) and Origin 21.0 (Origin 21.0, OriginLab Corp., Northampton, MA, USA).

*RI* and *SE* were calculated following Bruce and Richardson [55], as follows:

(1) $$RI=1-\frac{C}{T}\left(T\geq C\right),RI=\frac{T}{C}-1(T<C)$$

where *T* and *C* represent the test and control group data, respectively. The *RI* ranged from −1 to +1, with positive values indicating stimulation and negative values indicating treatment-mediated inhibition of seedling growth compared with the controls [55].

(2) $$SE=\frac{\left(RI1+RI2+\cdots +Rin\right)}{n}$$

Here, *n* is the number of *RI*s, and *SE* indicates the effect of allelopathy. *SE* was applied to evaluate the average allelopathic effects of several *RI*s [56].

## 5. Conclusions

A comprehensive and systematic study was conducted on the physiological and molecular effects of *E. jolkinii* allelopathy, which inhibited *A. hookeri* seedling growth via multiple targets and pathways. These effects are responsible for the induction of a targeted plant hormone imbalance, inhibiting photosynthesis, and disrupting oxidation systems through allelopathy. However, to further elucidate the allelopathic effects of *E. jolkinii* and guide the management and control of its invasion, the analysis of specific allelochemicals is the future direction.

## Data Availability

Data inquiries can be directed to the corresponding authors.

## Supplementary Materials

The following supporting information can be downloaded at https://www.mdpi.com/article/10.3390/plants14010123/s1, Table S1: All metabolites in *Arundinella hookeri* seedlings; Table S2: All metabolites of *Arundinella hookeri* seedlings in volcano plots (control group vs. EJAE-40, supplementary materials of Figure 6a); Table S3: All metabolites of *Arundinella hookeri* seedlings in volcano plots (control group vs. EJAE-80, supplementary materials of Figure 6b); Table S4: All metabolites of *Arundinella hookeri* seedlings in volcano plots (control group vs. EJAE-120, supplementary materials of Figure 6c).
